# Low-Frequency Repetitive Transcranial Magnetic Stimulation over Right Dorsolateral Prefrontal Cortex in Parkinson's Disease

**DOI:** 10.1155/2020/7295414

**Published:** 2020-09-14

**Authors:** Sheng Zhuang, Fu-Yu Wang, Xin Gu, Jia-Jing Wu, Cheng-Jie Mao, Hao Gui, Jing Chen, Chun-Feng Liu

**Affiliations:** ^1^Department of Neurology and Suzhou Clinical Research Center of Neurological Disease, The Second Affiliated Hospital of Soochow University, Suzhou, China; ^2^Department of Neurology, Suqian First Hospital, Suqian, China; ^3^Jiangsu Key Laboratory of Neuropsychiatric Diseases and Institute of Neuroscience, Soochow University, Suzhou, China

## Abstract

**Background:**

Repetitive transcranial magnetic stimulation (rTMS) is a promising therapeutic tool for Parkinson's disease (PD), and many stimulation targets have been implicated. We aim to explore whether low-frequency rTMS over the right dorsolateral prefrontal cortex (DLPFC) improves motor and nonmotor symptoms of individuals with PD.

**Methods:**

We conducted a randomized, single-blind, sham-controlled parallel trial to compare the effect of 10 consecutive daily sessions of 1 Hz rTMS over right DLPFC on individuals with idiopathic PD between active and sham rTMS group. Primary outcomes were changes in Unified Parkinson's Disease Rating Scale (UPDRS) part III and Nonmotor Symptom Questionnaire (NMSQ). Secondary outcomes were changes in UPDRS total score, Hamilton Rating Scale for Depression (HRSD), Pittsburgh Sleep Quality Index (PSQI), and Montreal Cognitive Assessment (MoCA). Assessments were completed at baseline, after treatment, and at 1 month, 3 months, and 6 months after treatment.

**Results:**

A total of 33 participants with PD were randomized. All participants completed the study and no severe adverse effect was noticed. Compared to baseline, active rTMS showed significant improvements in UPDRS part III and NMSQ at 1 month. Change of scores on UPDRS part III, HRSD, and PSQI persisted for 3 months after rTMS intervention. The beneficial effect on cognitive performance assessed by MoCA was maintained for at least 6 months in the follow-up. No significant changes were observed in the group with sham rTMS.

**Conclusions:**

Low-frequency rTMS of right DLPFC could be a potential selection in managing motor and nonmotor symptoms in PD.

## 1. Introduction

Parkinson's disease (PD) is a second common neurodegenerative disease characterized by cardinal motor symptoms as bradykinesia, resting tremor, rigidity, and postural instability and gait disorders [1]. With pathology of widespread *α*-synuclein accumulation, various nonmotor symptoms such as depression, cognitive impairment, and sleep disturbances are involved [2]. A combination of these manifestations makes it thorny in management and adds much burden to individuals with PD and their caregivers [1, 2].

Repetitive transcranial magnetic stimulation (rTMS) is a promising noninvasive brain modulation technique in improving motor and nonmotor symptoms of PD in addition to pharmacological treatment [3, 4]. Although high-quality evidence for rTMS in the clinical setting was limited, different cortical regions have been implicated in benefiting symptoms of PD [5, 6]. Among the targets, the right dorsolateral prefrontal cortex (DLPFC) has been applied to alleviate PD-related depression using low-frequency rTMS based on the rationale of imbalanced interregional activity (overactive right DLPFC and underactive left DLPFC) [7]. Besides, right DLPFC plays a crucial role in executive function [8] which may be reflected in cognitive change and motor-related dysfunction such as speech or limb movement in PD [8, 9]. Sleep disturbance, one common nonmotor symptom of PD, was also suggested to benefit from low-frequency rTMS over DLPFC [10]. However, previous studies investigating motor improvement with rTMS over DLPFC generated mixed findings [11]. Whether such stimulation was useful in dealing with other nonmotor symptoms (i.e., cognition and sleep) remained unknown in individuals with PD.

To address these gaps, we conducted a randomized, sham-controlled trial to explore the effect of a 10-day low-frequency rTMS over the right DLPFC on both motor symptoms and nonmotor symptoms of individuals with PD. We also evaluated the long-term therapeutic effect during a 6-month follow-up.

## 2. Methods

### 2.1. Study Design

This was a randomized, sham-controlled, single-blind, 2-arm parallel study to investigate the therapeutic effect of low-frequency rTMS over the right DLPFC in individuals with PD. This study was approved by the ethics committee of the Second Affiliated Hospital of Soochow University. Written informed consent was obtained from each participant prior to the study intervention.

### 2.2. Participants

We included participants with idiopathic PD who met the 2015 Movement Disorder Society diagnostic criteria for clinically established PD [12] from our movement disorder clinic between September 1, 2017, and February 1, 2018. Enrolled participants were assessed for eligibility on the following inclusion criteria: aged between 40 and 85 years old, right-handed Han Chinese, Hoehn and Yahr stage ≤3 during “ON” state, and stable dosage of anti-PD medications for at least 30 days from baseline and throughout the study period. Participants were excluded if they have a medical history of head trauma, stroke, epilepsy, psychiatric disorder, or severe cardiac disease, use recently relevant medications (i.e., benzodiazepines, antidepressants, or antipsychotic agents within 3 months), or are involved in any clinical trials within the past 6 months.

### 2.3. rTMS Protocol

Motor evoked potential (MEP) was recorded via electrodes over abductor pollicis brevis (APB). Resting motor threshold (RMT) refers to the minimum intensity to initiate at least 5 out of 10 consecutive MEPs over 50 *μ*V in relaxed APB muscle. In our study, a 70 mm diameter figure-of-8 coil was connected to the Magstim Super Rapid Stimulator (Magstim Ltd., UK). RMT was determined from the right primary motor cortex (M1), where MEP reached its maximal amplitude in the left APB. To locate the right DLPFC, the coil was moved 5 cm anterior to right M1. A red-colored dot was then spotted at the right DLPFC for subsequent stimulation.

In the active rTMS group, stimulation was delivered by a double-surface air-cooled coil connected to Magstim Super Rapid Stimulator (Magstim Ltd., UK). The surface of the coil was set tangentially to the scalp site with its handle pointing backward to the participant. We applied rTMS with 1 Hz, 1200 daily stimuli, 20 minutes per session, with an output stimulus intensity at 110% RMT. Each participant was administered rTMS at the same time of the day for 10 consecutive days. In the sham rTMS group, the stimulation coil was flipped over (180 degrees from original position) to provide identical sound and appearance and was only identified by physicians who conducted the therapy. The stimulation position and parameters were the same as those in the active group. To assess and enhance the adherence of participants, a biweekly telephone follow-up was arranged for the confirmation of general health status and informing participants about the date of evaluation.

### 2.4. Outcome Assessment

Unified Parkinson's Disease Rating Scale (UPDRS) part III was used to evaluate motor symptoms for individuals with PD. For nonmotor symptoms, Nonmotor Symptom Questionnaire (NMSQ), Hamilton Rating Scale for Depression-24 item (HRSD), Pittsburgh Sleep Quality Index (PSQI), and Montreal Cognitive Assessment (MoCA) were used to evaluate the overall nonmotor performance, depression, sleep quality, and cognition, respectively. Each participant was required to complete assessments at baseline, after treatment (immediately after the completion of all 10 sessions), and at 1 month, 3 months, and 6 months after the intervention. Evaluations were completed by well-trained physicians or movement disorder specialists. Primary outcomes were changes in UPDRS-III and NMSQ at 1 month after rTMS treatment. The secondary endpoint was the changes in UPDRS total score, PSQI, HRSD, and MoCA at all assessment time points of follow-up. Side effects were recorded during and after rTMS. All participants were assessed during the “OFF” state with at least 12 hours from the last use of anti-PD medication.

### 2.5. Randomization and Blindness

We used simple randomization to determine the assignment of each participant into two arms by flipping a coin (i.e., heads-sham group, tails-active group). Each participant was unaware of the allocation of the group and received rTMS in a separate room and time to avoid any conversation in between during the study period. Because physicians who delivered rTMS to certain participants may also be responsible for clinical assessment at a certain time point, we were unable to achieve complete blindness on investigators.

### 2.6. Statistical Analysis

Data analysis was performed from November 1, 2018, to December 1, 2018. SPSS 24.0 (SPSS Inc., USA) was used to perform statistical analysis, and two-sided *P* < 0.05 was established as the level of significance. Variable normality was tested by the Shapiro–Wilk method. Demographic and baseline clinical scores were analyzed using independent *t*-test, *χ*^2^ test, or Mann–Whitney *U* test. The significance of the outcome assessment was first evaluated by two-way repeated-measures analysis of variance (rANOVA) to evaluate the time course of change between active and sham rTMS groups. The Greenhous–Geisser coefficient was chosen for the adjustment of nonsphericity. Between the two groups, the analysis on scoring change at the same time was performed by an independent *t*-test. Within each group, a paired *t*-test was then used to compare the significance between every assessment point and baseline. The value of *α* was adjusted to 0.0125 (0.05/4) with Bonferroni correction for multiple comparisons.

## 3. Results

**Figure 1:**
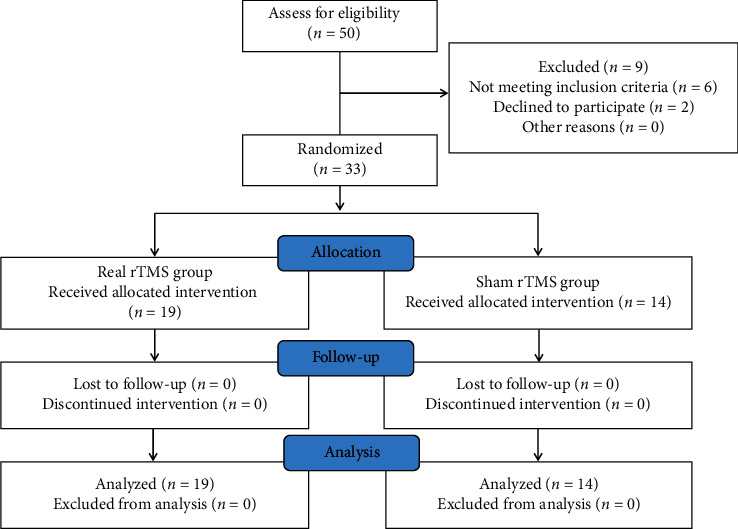
Study flow diagram.

A total of 50 participants with PD were assessed for eligibility and 33 of them (mean (SD) age: 61.0 (10.9) years; 18 (54.5%) male) were randomized (Figure 1). Participants were highly compliant with no dropouts during the study. At baseline, there were no significant differences in age, gender, disease duration and severity, levodopa equivalent dosage, and clinical assessment scores between two groups (Table 1).

**Figure 2:**
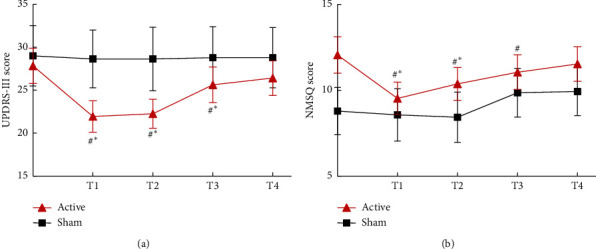
Score change of primary outcome on UPDRS part III and NMSQ in active and sham rTMS group.

After the intervention, a significant decrease in the UPDRS part III score was found in the active group but not in the sham group (−5.58 ± 3.37 points versus −0.36 ± 1.34 points, *P* < 0.001) in comparison with the baseline (Table 2; Figure 2). Absolute change for motor symptoms at 1 month after active rTMS was significant relative to sham stimulation (*P* < 0.001). NMSQ score at primary endpoint also showed significant improvement after active intervention but not after sham stimulation (−1.68 ± 2.11 points versus −0.36 ± 1.39 points, *P* < 0.001). Absolute change for the NMSQ score between active and sham groups, though not obvious to motor function, was statistically significant (Table 2; Figure 2).

In the secondary outcome, the time × group interaction was significant in UPDRS part III, HRSD, and MoCA scores (Table 3). After 10 consecutive sessions of active rTMS, UPDRS-III score experienced a significant downward trend with statistical significance after treatment and at 1 month and 3 months after treatment, suggesting an improvement of motor performance. By assessing UPDRS total scores, however, the overall therapeutic effect seemed to exist only for 1 month (Table 3). For nonmotor symptoms, the NMSQ score was reduced at the first two time points compared to baseline. In the 3-month visit, the reduction changes were no longer significant from pretreatment but were still significant when comparing to the sham group. As for HRSD, l Hz rTMS on the right DLPFC was useful to alleviate depression and the effect was maintained for at least 3 months, with maximum reduction points at 4.47 (Table 3). In the active group, the effect of rTMS on sleep quality, as assessed by PSQI, was not immediate after intervention but became statistically significant at 1 month and 3 months. To be noted, the MoCA score was unexpectedly improved at all study time points between groups as well as in comparison with baseline. The lasting effect on cognitive improvement was maintained for at least 6 months after rTMS intervention (Table 3).

For safety, the current rTMS protocol was overall well-tolerated by all participants. Two female participants in the active group reported transient mild headache during stimulation but were relieved soon. No severe adverse events were noticed.

## 4. Discussion

In this single-blind, sham-controlled, randomized trial, we reported that a 10-day consecutive 1 Hz rTMS over right DLPFC promoted motor and several nonmotor symptoms among individuals with PD. We also noticed that the current protocol had a sustained effect on cognitive improvement for individuals with PD.

DLPFC has emerged as one of the stimulation targets of interest in previous rTMS studies of PD. Neuroimaging studies revealed that hypoactivated left DLPFC was mainly associated with mood changes in PD [7]. High-frequency rTMS over left DLPFC, with comparable effects to those of antidepressants [13], was proved beneficial in treating PD-related depression in several studies [7, 14, 15]. Studies targeting the right DLPFC were largely based on its role of executive function such as working memory, decision making, and coping with novel tasks [8], which were found to be impaired at the early stage of PD [16]. Unilateral right DLPFC stimulation was shown to have a positive effect for individuals with PD on timed up-and-go task performance [17], spatial planning [18], metaphor comprehension [19], and time perception [20] via rTMS or other brain stimulation tools.

In the current study, we chose low-frequency rTMS over the right DLPFC for several reasons. First, relative to left DLPFC, executive dysfunction of right DLPFC was less discussed but could be reflected in movement reaction or presented on emotional and cognitive improvement after transcranial direct current stimulation [21]. Second, common sleep disturbance of PD, such as insomnia, was affected more preferentially in the right DLPFC-lateralized pattern [10]. However, the administration of rTMS on the right DLPFC in treating this nonmotor symptom was rarely explored in individuals with PD. Third, there have been few studies showing improvement of motor symptoms after rTMS to the right DLPFC while results were not consistent [11]. Fourth, low-frequency rTMS might be a safer option and better tolerated by individuals with PD compared with high-frequency rTMS.

Whether rTMS over DLPFC benefited motor symptoms was unsettled mainly because of various rTMS parameters (e.g., target selection, frequency, intensity, and total stimulus) and study participants. In the current trial, active rTMS group showed significant improvement in motor performance, which was similar to results from some meta-analyses [11, 22] examining the efficacy of either low frequency over right DLPFC or high frequency over left DLPFC on the motor symptom. Although the exact mechanisms remained elucidated, a reasonable explanation might be due to the release of dopamine in the striatum resulting from sustained stimulation on DLPFC via the frontal-striatal-cortical pathways [23, 24] in promoting global motor performance. In addition, the impaired executive function of DLPFC was associated with freezing of gait [25], and stimulation over the prefrontal cortex can modify gait abnormality in individuals with PD [26, 27]. That is to say, the observed UPDRS score change may be partially attributed to gait improvement. However, one should be aware that the conclusion and aforementioned mechanisms were all from studies using high-frequency rTMS, thus not directly supporting our findings. Whether the influence on motor severity was mediated by the improvement of depression remained to be addressed in further analysis.

As for nonmotor symptoms, a significant decrease in the NMSQ score was found at least 3 months after stimulation, which, to our knowledge, was not reported in initial studies. Findings from the secondary endpoint suggested that current stimulation protocol alleviated depressive symptoms in individuals with PD, which aligned well with previous studies showing the beneficial effect of low-frequency rTMS over right DLPFC in individuals with the major depressive disorder [28] or PD-related depression [11, 29]. It is believed that low-frequency rTMS to the hyperactivated right DLPFC can suppress the excitability of cortex and then leads to transsynaptic activation of the hypoactivated left DLPFC by reducing negative moods [30]. Of note, the cognitive performance also improved and had persisted up to 6 months after the intervention, which was similar to the beneficial long-term effect in cognitive treatment using transcranial direct current stimulation over DLPFC in individuals with PD [21]. Using rTMS, Patel et al. found that either excitatory or inhibitory stimulation over DLPFC had an insignificant influence on cognitive function in healthy adults [31]. However, such conclusion has not been examined in individuals with PD. In the current study, as observed on motor performance, the beneficial effect on cognition could be due to the improvement of depression because the two entities were commonly concurrent in individuals with PD [32] and both symptoms had shared neural pathway abnormality in frontostriatal circuitry [24]. Additionally, rTMS may have directly modulated the executive function center of PD by contributing to the improvement of selective domains in MoCA.

The strengths of our study included a randomized, sham-controlled design to explore the therapeutic effects of rTMS on individuals with PD with high compliance. Several validated questionnaires were used to assess both motor and nonmotor symptoms, and multiple evaluations in the study period enabled us to observe the short- and long-term therapeutic effects of rTMS. Several limitations should be kept in mind when interpreting our findings. First, the single-blind design may cause bias as we may have overestimated or underestimated the authentic effect of rTMS in the active and sham groups, respectively. However, we assigned different well-trained physicians for evaluations in the follow-up attempting to minimize the potential influence. Second, it was suggested that PD-related mood changes and cognitive impairment were associated with the underlying shared neural pathways [24]. Motor performance can also be influenced by negative emotions. Whether the improvement of motor or cognitive performance was attributed to the alleviation of depression cannot be inferred based on the current analysis. However, we observed that the beneficial effect on MoCA score persisted up to 6 months of the visit while significant changes on HRSD existed only in the first month of follow-up, which may partially suggest that the therapeutic influence of the right DLPFC rTMS on cognition might be independent. Third, accurate location is another concern because the determined cite using the conventional method (as was in our study, using 5 cm anterior to M1 as the markers for DLPFC) might not capture the desired stimulation cortex [33]. This could potentially affect the between-subject variability of therapeutic effects produced by rTMS. Future studies with MRI-guided navigation might be an optimal solution. Fourth, the sample size was quite small, and we did not calculate the subscore on different domains of our clinical scales in this preliminary study, which precluded us from obtaining further refined results. Finally, the generalizability was open to discussion as our results were from a single-centered trial.

## 5. Conclusion

This randomized, sham-controlled study indicated that low-frequency rTMS over the right DLPFC might be a potential treatment option for improving motor symptoms, depression, and cognitive performance in individuals with PD. Future studies with better designs are needed to confirm our findings, explore the biological mechanisms, and optimize tailored rTMS therapeutic protocols for individuals with PD.

## Figures and Tables

**Table 1:** Demographic and baseline characteristics of participants.

	Active group (*n* = 19)	Sham group (*n* = 14)	*P* value
Age, *y*	60.58 (9.21)	61.57 (13.25)	0.80
Gender, F/M	8/11	7/7	0.65^a^
Disease duration, *m*	70.37 (52.26)	68.57 (45.29)	0.91
Educated year, *y*	8.53 (2.07)	7.71 (2.27)	0.29^a^
H-Y stage	2 (1.5,2.5)	2.25 (1.75, 3.0)	0.38^b^
LED, mg	473.94 (214.79)	516.07 (210.22)	0.58
UPDRS part III	27.84 (8.96)	29.00 (13.16)	0.77
UPDRS	48.58 (15.90)	43.71 (19.13)	0.43
NMSQ	12.05 (4.61)	8.79 (5.16)	0.07
HRSD	13.26 (6.90)	15.86 (7.12)	0.30
PSQI	9.63 (4.87)	7.57 (3.25)	0.18
MoCA	24.37 (3.51)	22.64 (3.15)	0.16

Data are presented as mean (SD) or median (*Q*_25_, *Q*_75_). Abbreviations: H-Y stage: Hoehn and Yahr stage; LED: levodopa equivalent dose; UPDRS: Unified Parkinson's Disease Rating Scale; NMSQ: Nonmotor Symptom Questionnaire; HRSD: Hamilton Rating Scale for Depression; PSQI: Pittsburgh Sleep Quality Index; MoCA: Montreal Cognitive Assessment. ^a^*χ*^2^ test; ^b^Mann–Whitney *U* test.

**Table 2:** Primary outcome comparisons between active and sham rTMS groups.

	Group	Baseline score	Score at 1 month	Absolute change in score	*P*1	*P*2
UPDRS part III	Active	27.84 ± 8.96	22.26 ± 7.32	−5.58 ± 3.37	<0.001	<0.001
	Sham	29.00 ± 13.16	28.64 ± 13.84	−0.36 ± 1.34	0.34	
NMSQ	Active	12.05 ± 4.61	10.37 ± 4.18	−1.68 ± 2.11	<0.01	<0.05
	Sham	8.79 ± 5.16	8.43 ± 5.46	−0.36 ± 1.39	0.36	

Abbreviations: UPDRS: Unified Parkinson's Disease Rating Scale; NMSQ: Nonmotor Symptom Questionnaire. *P1*: paired *t*-test value between baseline and 1 month. *P*2: independent *t*-test between groups.

**Table 3:** Secondary outcome comparisons between active and sham rTMS groups.

		UPDRS part III	UPDRS	NMSQ	HRSD	PSQI	MoCA
Active	T0	27.84 (8.96)	48.58 (15.90)	12.05 (4.61)	13.26 (6.90)	9.63 (4.87)	24.37 (3.53)
	T1	21.95 (7.99)^ab^	39.32 (12.50)^ab^	9.53 (4.14)^ab^	8.79 (5.19)^ab^	7.63 (4.42)^b^	26.37 (2.67)^ab^
	T2	22.26 (7.32)^ab^	39.47 (11.44)^ab^	10.37 (4.18)^ab^	10.32 (5.33)^ab^	7.11 (3.91)^ab^	26.58 (3.67)^ab^
	T3	25.68 (8.58)^ab^	43.05 (14.82)	11.05 (4.4)3^b^	12.32 (6.47)^a^	7.3 (3.89)^ab^	26.58 (3.04)^ab^
	T4	27.00 (9.46)	44.26 (14.81)	11.53 (4.41)	12.74 (6.99)	8.00 (4.11)	26.47 (3.17)^ab^

Sham	T0	29.00 (13.16)	43.71 (19.13)	8.79 (5.16)	15.86 (7.12)	7.57 (3.25)	22.57 (3.13)
	T1	28.64 (12.57)	43.43 (18.47)	8.57 (5.69)	15.50 (6.78)	7.50 (3.06)	22.93 (2.92)
	T2	28.64 (13.84)	43.43 (19.86)	8.43 (5.46)	15.57 (7.23)	7.57 (3.13)	22.71 (2.97)
	T3	28.79 (13.43)	43.71 (21.36)	9.86 (5.33)	15.36 (6.92)	8.36 (3.95)	22.29 (2.87)
	T4	28.79 (13.11)	43.57 (21.18)	9.93 (5.26)	15.36 (7.22)	8.43 (3.84)	22.14 (2.91)

^*∗*^ *F*	14.215	3.153	3.461	13.916	5.052	9.192	
^*∗*^ *P*	<0.001	0.057	0.038	<0.001	0.006	<0.001	

Abbreviations: H-Y stage: Hoehn and Yahr stage; LED: levodopa equivalent dose; UPDRS: Unified Parkinson's Disease Rating Scale; NMSQ: Nonmotor Symptom Questionnaire; HRSD: Hamilton Rating Scale for Depression; PSQI: Pittsburgh Sleep Quality Index; MoCA: Montreal Cognitive Assessment. T0: baseline; T1: after treatment; T2: 1 month; T3: 3 months; T4: 6 months. ^*∗*^*F* and *P* values for rANOVA interaction (time and group) with adjustment for nonsphericity. ^a^Significant difference from baseline. ^b^Significant difference between groups.

## Data Availability

The data used to support the findings of this study are available from the corresponding author upon request.
